# *Ganoderma lucidum* Ameliorates Non-Alcoholic Steatosis by Upregulating Energy Metabolizing Enzymes in the Liver

**DOI:** 10.3390/jcm7060152

**Published:** 2018-06-15

**Authors:** Soonwoong Jung, Hyeonwi Son, Chung Eun Hwang, Kye Man Cho, Sang Won Park, Hyun Joon Kim

**Affiliations:** 1 Bio Anti-Aging Medical Research Center, Department of Anatomy and Convergence Medical Science, Institute of Health Sciences, Gyeongsang National University School of Medicine, Jinju 52727, Korea; birth1110@gnu.ac.kr (S.J.); haeny@gnu.ac.kr (H.S.); 2 Department of Food Science, Gyeongnam National University of Science and Technology, Jinju 52725, Korea; poponot@naver.com (C.E.H.); kmcho@gntech.ac.kr (K.M.C.); 3 Bio Anti-aging Medical Research Center, Department of Pharmacology and Convergence Medical Science, Institute of Health Sciences, Gyeongsang National University School of Medicine, Jinju 52727, Korea

**Keywords:** *Ganoderma lucidum*, high-fat diet, insulin resistance, non-alcoholic fatty liver, obesity

## Abstract

Non-alcoholic steatosis is a common health problem worldwide due to altered food habits and life styles, and it is intimately linked with various metabolic disorders. In the present study, we investigated the molecular mechanism of *Ganoderma lucidum* (GL) against the development of non-alcoholic steatosis using in vivo and in vitro settings. C57BL/6 mice fed with normal diet (ND) or high fat diet (HFD) were administered GL extract or vehicle for 16 weeks. HFD feeding increased serum alanine aminotransferase level and hepatic lipid droplet, but these increases were significantly attenuated by GL. GL inhibited the increases in epididymal and perirenal adipose tissue weights and serum cholesterol and LDL levels in HFD-fed mice. Fasting blood glucose levels were elevated in HFD-fed mice compared to ND-fed mice, and glucose and insulin sensitivities were deteriorated. These changes were markedly improved by GL. GL restored the reduction of AMP-activated protein kinase (AMPK) and acetyl-CoA carboxylase (ACC) phosphorylation in the liver of HFD-fed mice, and increased AMPK and ACC phosphorylation in HepG2 and 3T3-L1 cells. GL induced GLUT4 protein expression in 3T3-L1 cells. Finally, GL attenuated lipid accumulation induced by free fatty acid in HepG2 cells. Taken together, our results indicate that GL has a potential to improve non-alcoholic steatosis and the associated complicated disorders via the induction of energy metabolizing enzymes.

## 1. Introduction

Nonalcoholic fatty liver disease (NAFLD) is considered to be a pathological condition being linked to the development of numerous health problems and the diminishment of life expectancy. NAFLD is closely related to obesity and dyslipidemia, which ultimately contributes to insulin resistance, type 2 diabetes [1,2]. The prevalence of obesity is continuously increasing worldwide and becomes a major threat to public health [3].

The early stage of NAFLD is the nonalcoholic fatty liver (simple steatosis), which is accompanied by excessive lipid or triglyceride (TG) deposition in hepatocyte without the initiation of inflammation. Since it is reversible to the normal condition, non-alcoholic steatosis is the optimal therapeutic stage of NAFLD. Previous studies revealed that hepatic TG content is obtained 60% from free fatty acids (FFAs) of adipose tissues, 26% from de novo lipogenesis, and 15% from the diet in steatotic patients [4]. In healthy individuals, de novo lipogenesis contributes only <5% of their hepatic TG [5]. Hence, de novo lipogenesis and FFAs are key factors for the development of non-alcoholic steatosis.

*Ganoderma lucidum* (GL), known as different names in China, Japan and Korea, is a *Basidomycete* white root fungus, and has been used for eras to improve health and longevity [6]. A previous study showed that GL contains flavonoids and polyphenolics, such as chlorogenic acid, caffeic acid, quercertin, and kaempferol [7]. Fractions extracted from GL were effective against cancer, diabetes, and hepatotoxicity [8]. GL induced cell-cycle arrest and apoptosis in various human and rodent tumor cells, including human hepatoma Huh-7 cells, where GL prolonged the G2 cell cycle, resulting in strong growth inhibition [9]. GL was also found to lower the serum glucose levels in diabetic (+db/+db) mice via hepatic phosphoenolpyruvate carboxykinase gene regulation [10]. In addition, GL was hepatoprotective against d-galactosamine-induced liver injury in mice [11]. Although GL exhibits a wide range of therapeutic effect, the mechanical study of GL against non-alcoholic steatosis remains to be unveiled. In the present study, we investigated the effect of GL extract on the development of non-alcoholic steatosis using in vitro and in vivo experimental models.

## 2. Materials and Methods

### 2.1. Preparation of GL Extract

GL was extracted according to a previous study [12]. Dried GL was produced by Imsil Lingzi mushroom farming association corporation (Jeonbuk, Korea). GL extract was prepared by Kye Man Cho using ethanol extraction method. Ten grams of dried GL was mixed with 200 mL of 70% ethanol, and was extracted by centrifugation at 600 rpm, 40 °C for 4 h. The extraction was repeated two times, and the extracts from three extractions were combined and filtered using the filter paper. The filtered extract was concentrated at 60 °C. GL extract was dissolved in the water including 0.5% Tween-20, and sonicated for 1 h. Final stock solution of GL extract was 5 mg/mL of concentration. Extraction setting was optimized by a preliminary study, which determined the amount of beta-glucan and vitamin B (Supplementary Materials).

### 2.2. Animals and Treatments

Male C57BL/6 mice (three weeks old) were purchased from KOATECH (Pyeongtaek, Korea) and maintained in the animal facility at Gyeongsang National University. The experiments were performed in accordance with the National Institutes of Health Guidelines on the Use of Laboratory Animals. The university animal care committee for animal research of Gyeongsang National University approved the study protocol (GNU-141119-M0055). Mice were housed with a 12-h light/dark cycle at 25 °C and allowed free access to water and normal laboratory diet for one week before being divided randomly into four experimental groups (10–11 mice per group). The mice fed with normal diet (ND; fat 6% of total kcal) and high fat diet (HFD; fat 45% of total kcal) (Research Diets, Inc., New Brunswick, NJ, USA) were orally administered with GL (50 mg/kg) in sterilized water five days/week for 16 weeks. This dose was selected because it has been evaluated previously [10]. Food intake and body weight were monitored twice in a week throughout the period of experiment. After 16 weeks of HFD feeding, the mice were anesthetized under CO_2_ inhalation, and then removed and froze the tissues very quickly.

### 2.3. Measurement of Blood Glucose Level

Fasting blood glucose level was measured in the mice fasted for 16 h (overnight) at 9:30 a.m. once in every two weeks. A drop of blood was taken from the tail vein and blood glucose level was measured using an Optium Xceed glucometer (Abbott, Abbott Park, IL, USA).

### 2.4. Glucose Tolerance Test and Insulin Tolerance Test

Mice were fasted for 16 h before the glucose tolerance test (GTT) and insulin tolerance test (ITT). d-glucose (2 g/kg) was intraperitoneally injected and blood samples were taken from the tail vein before and 30, 60, 90, and 120 min after the injection of glucose. Blood glucose level was measured by glucometer. To perform ITT, mice were injected with 0.1 mL of 0.9% normal saline containing insulin (1 U/kg, Humulin-R; Lilly USA, LLC, Indianapolis, IN, USA). A drop of blood was taken from the tail vein before and 30, 60, 90, and 120 min after the injection of insulin, and blood glucose level was measured by glucometer.

### 2.5. Biochemical Assay

Serum alanine aminotransferase (ALT), aspartate aminotransferase (AST), alkaline phosphatase (ALP), total cholesterol (TC), triglyceride (TG), low density lipoprotein (LDL) and high-density lipoprotein (HDL) levels were determined using enzymatic colorimetric assays from Green Cross Reference Laboratory (Yongin-si, Korea).

### 2.6. H&E and Oil Red O Stain in Liver Tissue

The mice were perfused with 4% paraformaldehyde for histological examination. Liver (5 μm) sections were stained with H&E by standard methods [13]. Frozen liver sections (10 μm) were stained with 0.3% Oil Red O in 60% isopropanol for 40 min and were counterstained with hematoxylin. The cells were washed, fixed with 10% PBS-buffered formalin for 1 h at room temperature, and then stained with 2.1 mg/mL Oil Red O in 60% isopropanol for 10 min. The images were obtained under a BX51 light microscopy. Oil Red O was eluted by adding 100% isopropanol, and then the quantity was measured at 545 nm using a microplate reader (Infinite F200, Tecan Group Ltd., Männedorf, Switzerland). Steatosis score was graded on a scale of 0 (<5%), 1 (5–33%), 2 (34–66%) and 3 (>66%) according to the previous study [14].

### 2.7. Western Blot Analysis

Cells or liver tissues were collected and frozen quickly to avoid degradation or dephopsphorylation. The samples were homogenized in a RIPA lysis buffer including protease and phosphatase inhibitors and incubated on ice for 20 min. The supernatant was collected by centrifuging at 14,000 *g* at 4 °C for 10 min. Protein samples were separated with SDS-PAGE, and then transferred to Polyvinylidene fluoride membranes (Roche, Mannheim, Germany). The membranes were incubated to the primary antibodies and respective secondary antibodies. Primary antibodies for phosphorylated adenosine monophosphate activated kinase (pAMPK, #2535), AMPK (#2532), phosphorylated acetyl-CoA carboxylase (pACC, #3661), and ACC (#3662) were purchased from Cell Signaling Technology. Primary antibodies for glucose transporter 4 (GLUT4) (ab654) and β-actin (A2228) were purchased from Abcam (Cambridge, UK) and Sigma-Aldrich (Saint Louis, MO, USA), respectively. The binding of all antibodies was detected using an ECL detection system (Pierce, Rockford, IL, USA) according to the manufacturer’s instructions. The Multi-Gauge Version 3.0 image analysis program (Fujifilm, Tokyo, Japan) was used to measure band densitometry.

### 2.8. Cell Culture and GL Treatment

Human hepatoma HepG2 and pre-adipocyte 3T3-L1 cells were purchased from the American Type Culture Collection (Manassas, VA, USA) and maintained in low-glucose Dulbecco’s modified Eagle’s medium (Invitrogen, Grand Island, NY, USA) supplemented with 10% (*v*/*v*) heat-inactivated FBS, penicillin G (100 U/mL), streptomycin (100 mg/mL), and L-glutamine (2 mM) at 37 °C in 5% CO_2_. After reaching 75% confluence, the cells were starved for 16 h, and then exposed to an FFA mixture to induce fat overloading of the cells. An FFA mixture at a 2:1 ratio of oleate/palmitate was diluted in culture medium containing 1% fatty acid-free bovine serum albumin to reach the desired final concentrations [15]. The control cells were treated with 1% bovine serum albumin.

### 2.9. Cytotoxicity of GL

Twenty-four hours after treatment with the GL or FFA mixture, the culture mediums were collected to measure the released lactate dehydrogenase (LDH) level. LDH level was measured using CytoTox 96^®^ Non-Radioactive Cytotoxicity Assay kit (Promega, Madison, WI, USA) according to the manufacturer’s instruction. Cell viability was also measured using the 3-(4,5-dimethylthiazol-2-yl)-2,5-diphenyltetrazolium bromide (MTT) cell proliferation assay. One mg/mL MTT solution was added to each well and incubated at 37 °C in 5% CO_2_ for 3 h. The medium was removed, and DMSO was added to dissolve the MTT-formazan complex. The absorbance was measured at 570 nm using a microplate reader.

### 2.10. Statistical Analysis

Statistical analysis was performed using the GraphPad Prism 5 (La Jolla, CA, USA). Statistical difference among the groups was determined with one-way analysis of variance (ANOVA), followed by Bonferroni post-hoc analysis. The values are expressed as the mean ± SEM. A *p* value < 0.05 was considered statistically significant.

## 3. Results

### 3.1. GL Attenuated the Increases in Liver and Adipose Tissue Weights in HFD-Fed Mice

**Figure 1:**
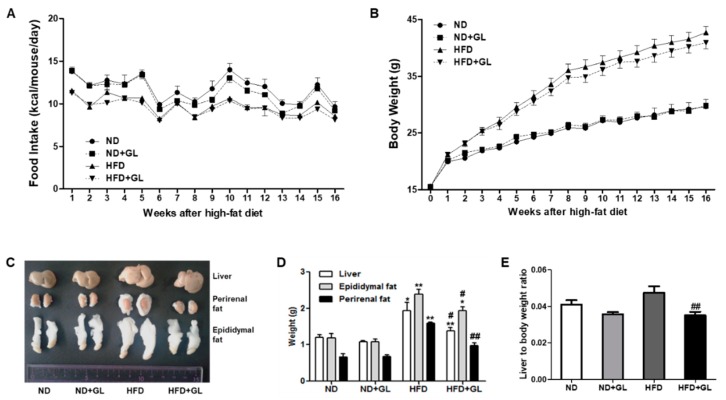
*Ganoderma lucidum* (GL) attenuated the increase in liver weight and epididymal and perirenal fat accumulation in high fat diet (HFD)-fed mice. (**A**) Food intake and (**B**) body weight for 16 weeks. (**C**) Representative images of liver, perirenal fat and epididymal fat and (**D**) the changes of the weight of liver, epididymal fat, and perirenal fat. (**E**) The ratio of liver weight to body weight. GL (50 mg/kg) or vehicle was orally administered five days/week in the mice fed with normal diet (ND) or HFD. Food intake was measured every ten days for 16 weeks. The mice were sacrificed after 16 weeks of GL treatment and the tissues were weighed (*n* = 8–9 for each group). Data are the mean ± SEM. * *p* < 0.05 and ** *p* < 0.01 vs. ND group; ^#^ *p* < 0.05 and ^##^ *p* < 0.01 vs. HFD group.

HFD feeding for 16 weeks significantly increased liver weight and epididymal and perirenal fat bodies compared to ND feeding, but these increases were attenuated by GL (Figure 1A,B). In addition, GL extract significantly reduced the liver to body weight ratio in HFD-fed mice (Figure 1C). GL did not affect food intake in both ND-fed and HFD-fed mice, indicating that the effect of GL on lipid accumulation was not due to the reduction of food intake (Figure 1D). 

### 3.2. GL Reduced Insulin Resistance in HFD-Fed Mice

**Figure 2:**
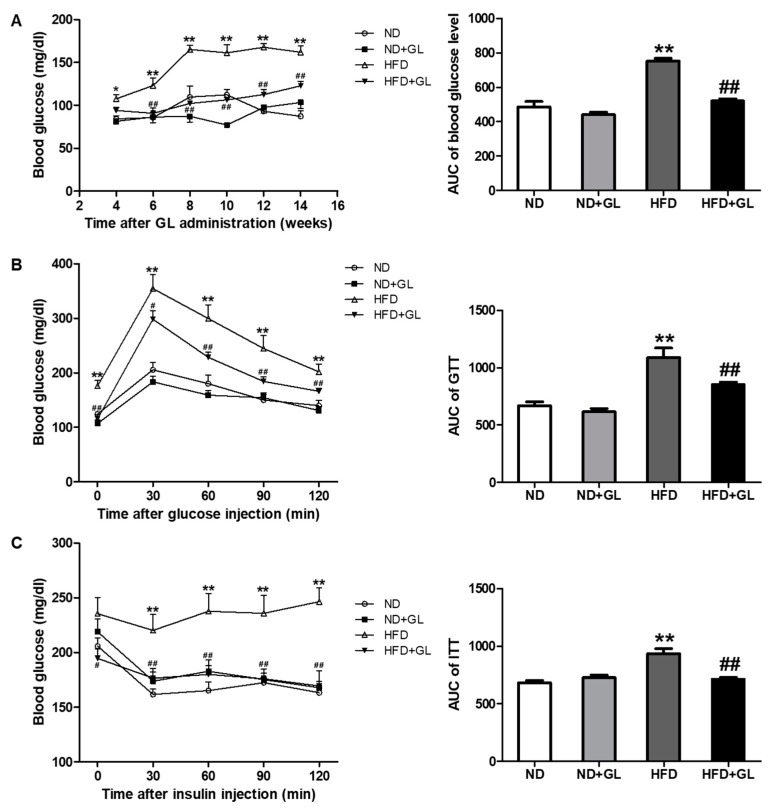
GL reduced fasting blood glucose level, glucose tolerance and insulin tolerance in HFD-fed mice. GL (50 mg/kg) or vehicle was orally administered five days/week in the mice fed with normal diet (ND) or high fat diet (HFD). The mice were fasted for 16 h to measure blood glucose level once in every two weeks. To conduct GTT and ITT, the mice were fasted for 16 h at 14 weeks of GL treatment. (**A**) Fasting blood glucose level. (**B**) Glucose tolerance test was performed by injecting 20% d-glucose (2 g/kg body weight). (**C**) Insulin tolerance test was performed by injecting insulin (1 U/kg body weight). *n* = 8–9 for each group. Data are the means ± SEM. * *p* < 0.05 and ** *p* < 0.01 vs. ND group; ^#^ *p* < 0.05 and ^##^ *p* < 0.01 vs. HFD group.

To determine the anti-diabetic effect of GL extract, fasting blood glucose level, GTT and ITT were analyzed. Fasting blood glucose levels were measured every two weeks. HFD-fed mice significantly increased fasting blood glucose level compared to ND-fed mice, but these increases were inhibited from six weeks of GL treatment (Figure 2A). GTT and ITT were measured at 14 weeks after GL administration. GL treatment significantly reduced the increases in both GTT and ITT in HFD-fed mice (Figure 2B,C).

### 3.3. GL Reduced Serum Total Cholesterol and Low-Density Lipoprotein in HFD-fed Mice

**Figure 3:**
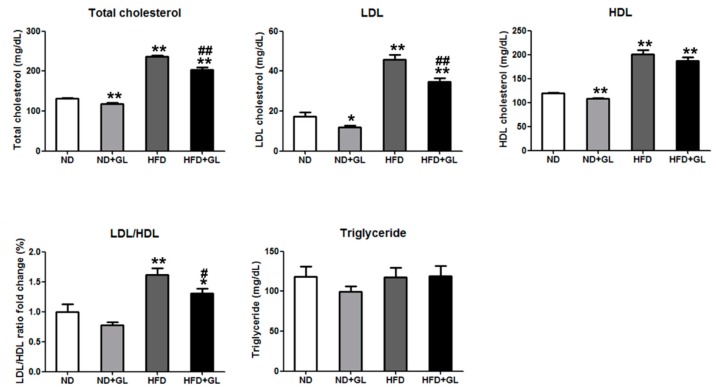
GL attenuated the increases in serum cholesterol and low-density lipoprotein (LDL) levels in HFD-fed mice. GL (50 mg/kg) or vehicle was orally administered five days/week in the mice fed with normal diet (ND) or high fat diet (HFD). Serum were collected to measure total cholesterol, triglyceride, LDL, and HDL levels after 16 weeks of GL treatment (*n* = 8–9 for each group). Data are the means ± SEM. * *p* < 0.05 and ** *p* < 0.01 vs. ND group; ^#^ *p* < 0.05 and ^##^ *p* < 0.01 vs. HFD group.

Serum total cholesterol, LDL and HDL levels considerably increased in HFD-fed mice compared to ND-fed mice. GL significantly reduced the increases in serum total cholesterol, LDL, and LDL/HDL levels in HFD-fed mice, whereas did not affect serum HDL level. TG levels were not different in all group of mice (Figure 3).

### 3.4. GL Attenuated Serum ALT Level and Hepatic Lipid Accumulation in HFD-fed Mice

**Figure 4:**
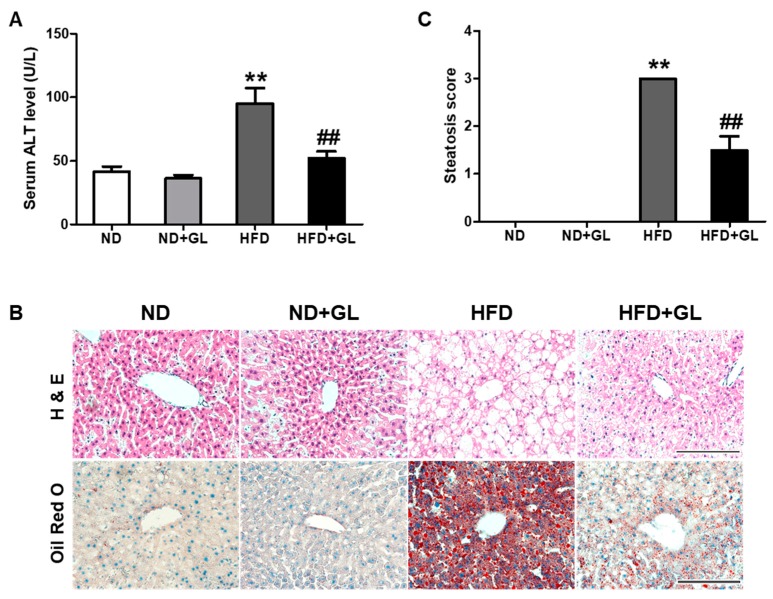
GL attenuated hepatic lipid accumulation and the increase in serum alanine aminotransferase (ALT) level in HFD-fed mice. (**A**) Serum alanine aminotransferase level (*n* = 8–9 for each group). (**B**) Representative microphotographs (Scale bar = 100 µm) of hematoxylin and eosin-stained and Oil Red O-stained liver sections (*n* = 5–6 for each group). (**C**) Steatosis score estimated from H&E slides for each group. GL (50 mg/kg) or vehicle was orally administered five days/week in the mice fed with normal diet (ND) or high fat diet (HFD). The serum and liver tissues were collected after 16 weeks of GL treatment. Data are the means ± SEM. ** *p* < 0.01 vs. ND group; ^##^ *p* < 0.01 vs. HFD group.

HFD-fed mice had significantly elevated serum ALT levels compared to ND-fed mice, while GL significantly blocked the increase in serum ALT level (Figure 4A). H&E and Oil Red O staining presented the predominant macrovesicular steatosis in the liver tissue from HFD-fed mice. Hepatic lipid accumulation was markedly attenuated by GL (Figure 4B,C).

### 3.5. GL Increased Phosphorylation of AMPK and ACC in HFD-Fed Mice

**Figure 5:**
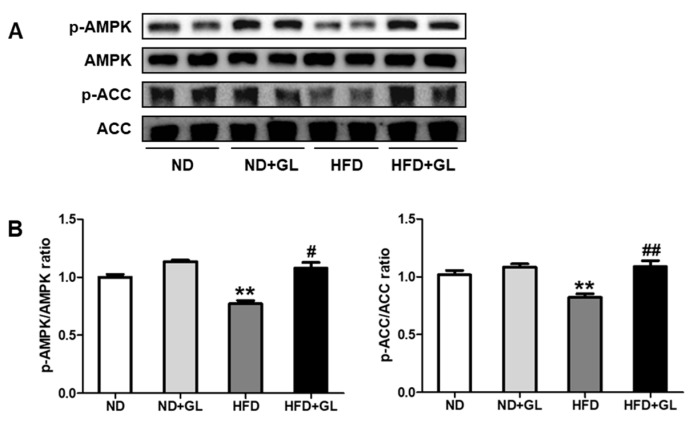
GL induced AMP-activated protein kinase (AMPK) and acetyl-CoA carboxylase (ACC) phosphorylation in HFD-fed mice. (**A**) Representative band images of Western blot analysis in the liver tissues (*n* = 5–6 for each group) and (**B**) densitometric quantifications of relative band intensities. GL (50 mg/kg) or vehicle was orally administered five days/week in the mice fed with normal diet (ND) or high fat diet (HFD). The liver tissues were collected after 16 weeks of GL treatment. Data are the means ± SEM. ** *p* < 0.01 vs. ND group; ^#^ *p* < 0.05 and ^##^ *p* < 0.01 vs. HFD group.

Due to the anti-steatotic effect of GL, we further elucidated the effect of GL on lipolytic enzymes (AMPK and ACC) in the liver. HFD feeding for 16 weeks reduced the phosphorylation of AMPK and ACC, but these were significantly attenuated by GL (Figure 5).

### 3.6. GL Increased Phosphorylation of AMPK and ACC and GLUT4 Expression in HepG2 and 3T3-L1 Cells

**Figure 6:**
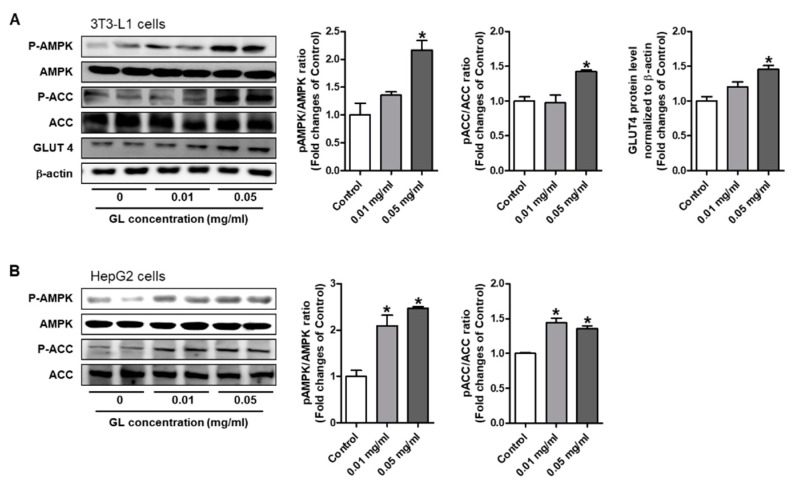
GL induced AMPK and ACC phosphorylation and GLUT4 protein expression in 3T3-L1 and HepG2 cells. Representative band images of western blot analysis and densitometric quantifications of relative band intensities in (**A**) 3T3-L1 and (**B**) HepG2 cells. GL (0.01 and 0.05 mg/mL) or vehicle was treated in the 3T3-L1 and HepG2 cells and protein levels were analyzed using Western blot. Data are the means ± SEM from three independent experiments. * *p* < 0.05 vs. control group (0 mg/mL concentration).

Next, we tested whether GL affected AMPK, ACC and GLUT4 protein expression in hepatocytes and adipocytes. GL significantly increased the phosphorylation of AMPK and ACC in HepG2 and 3T3-L1 cells. Moreover, GL also induced GLUT4 protein level in 3T3-L1 cells (Figure 6).

### 3.7. GL Attenuated Lipid Accumulation Induced by FFA in HepG2 Cells

**Figure 7:**
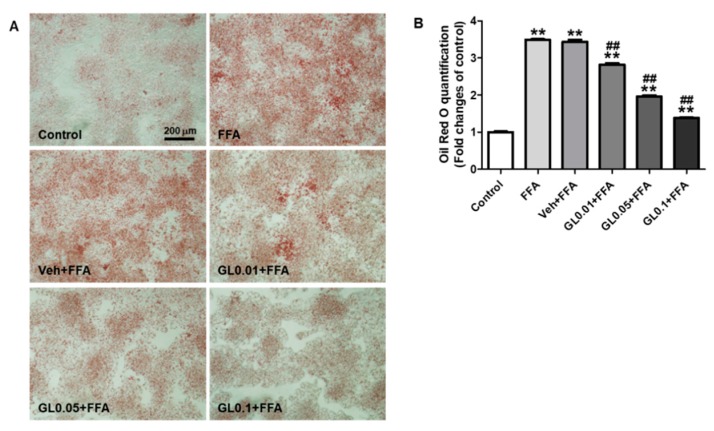
GL attenuated FFA-induced lipid accumulation in HepG2 cells. (**A**) Representative pictures (magnification 200×, scale bar = 200 μm) of Oil Red O stain in HepG2 cells from three independent experiments and (**B**) the quantification of lipid contents. HepG2 cells were treated with FFA (1 mM) for 24 h, and the cells were stained with Oil Red O dye. GL extract was pretreated 1 h prior to FFA treatment in the indicated concentrations, and control cells were treated with fatty acid-free BSA (1%). Data are the means ± SEM. ** *p* < 0.01 vs. control group; ^##^ *p* < 0.01 vs. FFA group.

To test whether GL had a direct effect on the lipid accumulation in hepatocyte, HepG2 cells were treated with 1 mM FFA for 24 h to induce the lipid accumulation. FFA exposure induced a 3.5-fold increase in lipid contents compared to control. However, this increase was significantly inhibited by GL extract in a concentration-dependent manner (Figure 7).

### 3.8. Cytotoxicity of GL Extract on HepG2 Cells

**Figure 8:**
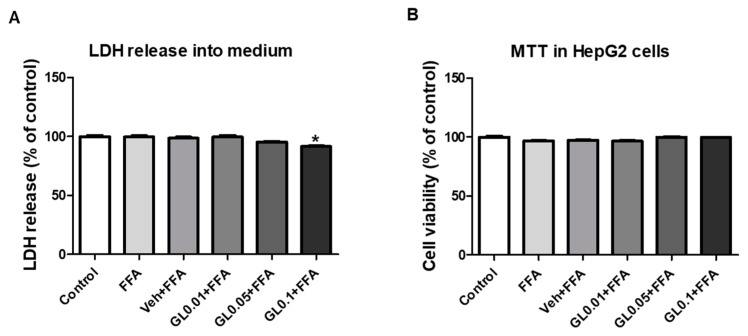
Cytotoxicity of GL in HepG2 cells. (**A**) LDH level in cultured medium. (**B**) MTT assay in HepG2 cells. HepG2 cells were treated with FFA (1 mM) for 24 h, and then cultured medium was collected for LDH assay and cells were harvested for MTT assay. GL extract was pretreated 1 h prior to FFA treatment in the indicated concentrations, and control cells were treated with fatty acid-free BSA (1%). Data are the means ± SEM from three independent experiments. * *p* < 0.05 vs. control group.

Because passive cell damage can cause a decrease in the number of Oil Red O-stained cells, we also determined the cytotoxicity of GL at the effective concentrations. To test the cytotoxicity of GL extract, we measured both extracellular LDH release and MTT-based cell viability. FFA, vehicle, or GL (up to 0.1 mg/mL) with FFA did not cause cytotoxicity (Figure 8).

## 4. Discussion

In the present study, we investigated the molecular mechanism of GL against non-alcoholic steatosis associated to obesity. HFD-fed mice developed the symptoms of non-alcoholic steatosis, indicated by the increase in liver/body weight ratio, hepatic fat droplets, and serum ALT level. However, these abnormalities were successfully ameliorated by GL treatment. Consistent with our results, a recent study showed that water extract of GL is highly effective to reduce obesity by modulating gut microbiota in rodents [3]. Other studies reported that GL fruit body extract is effective in treating obesity by regulating the expression of metabolic enzymes [16].

Hepatic insulin resistance was related to subcutaneous abdominal fat mass [17]. Increased cellular fatty acid derivatives activate stress kinases, led to phosphorylation of serine in insulin receptor substrate (IRS) proteins [18]. A clinical study revealed a strong association between insulin resistance and hepatic steatosis [18]. Compromised glucose tolerance is an indication of insulin insensitivity and abnormalities in glucose disposal [19,20]. GLUT4 plays a central role for the glucose transport in the muscle and fat tissues [21]. The translocation of glucose by GLUT4 is an insulin dependent process and also a rate limiting step for the utilization of glucose [21,22]. In this study, fasting blood glucose level started to reduce six weeks after GL treatment in HFD-fed mice, and glucose and insulin sensitivities were improved by GL treatment. In addition, GL induced GLUT4 protein level in adipocytes. These results present that GL can alleviate hyperglycemia through its anti-steatotic effect.

Serum lipid profile is a diagnostic and therapeutic target in the patients with metabolic disorder. In steatotic circumstances, excessively accumulated TG in hepatocytes is secreted as very low-density lipoprotein (VLDL). VLDL is an obligatory precursor of LDL, which has the atherogenic properties. It is widely appreciated that hepatic overproduction of VLDL contributes to the various hyperlipidemic states in humans, including familial combined hyperlipidemia [23] and diabetes [24]. In the present study, GL reduced serum TC and LDL levels in HFD-fed mice. Therefore, our data shows that GL may improve serum lipid profiles and inhibit the development of non-alcoholic steatosis.

Recently, a study using simple fat computed tomography reported that fat infiltration of the liver is correlated with amount of abdominal fat. Excessive fat in adipose tissues can increase circulating FFA leading to hepatic steatosis [25] and sixty percent of hepatic TG accumulation is resulted from FFA of adipose tissues. Our data showed that GL reduced the liver weight as well as the increases in perirenal and epididymal fat mass in HFD-fed mice. These results indicate that anti-steatotic effect of GL is partially due to the reduction of intraperitoneal fat mass.

A heterotrimeric serine-threonine kinase, AMPK, functions as a key cellular energy sensor in most tissues, and a potential therapeutic target for the prevention and/or treatment of NAFLD by many natural compounds [26]. AMPK acts as a critical node to connect insulin signaling and lipid metabolism and functions as a positive regulator of insulin resistance [15,26]. The net effect of AMPK is stimulation of hepatic fatty acid oxidation and ketogenesis, inhibition of cholesterol synthesis, lipogenesis, and TG synthesis [27]. Activation of AMPK results in inhibition of ACC that reduces production of malonyl-CoA which is a critical precursor for fatty acid synthesis and a potent inhibitor of fatty acid oxidation via carnitine palmitoyltransferase-1 in the liver [28]. In the present study, GL recovered the reduction of AMPK and ACC phosphorylation in the liver of HFD-fed mice. GL also induced the phosphorylation of AMPK and ACC in hepatocytes and adipocytes. In addition, GL attenuated the hepatic lipid accumulation induced by FFA. These results indicate that GL can regulate the lipid metabolism by activating lipolytic enzymes in the hepatocyte and adipocyte. 

## 5. Conclusions

Taken together, our results suggest that GL regulates the energy metabolizing process and lipid accumulation directly in the liver and adipocyte, and improves insulin sensitivity and the metabolic complications in a mouse model of diet-induced obesity. Therefore, GL is a potential candidate for preventing or treating NAFLD and metabolic disorders.

## Supplementary Materials

The following are available online at http://www.mdpi.com/2077-0383/7/6/152/s1, Table S1: β-Glucan and soluble vitamins contained in *Genoderma Lucidum* extract.
