# Supplementary material for: Ganoderma lucidum Ameliorates Non-Alcoholic Steatosis by Upregulating Energy Metabolizing Enzymes in the Liver
Source: J Clin Med. 2018 Jun 15;7(6):152. doi: 10.3390/jcm7060152 (PMC6025418; doi:10.3390/jcm7060152)
Supplement: Supplementary file 1 [file jcm-07-00152-s001.pdf]

Supplementary Materials: Ganoderma lucidum Ameliorates Non-Alcoholic Steatosis by Upregulating Energy Metabolizing Enzymes in the Liver

1.1. β-Glucan and soluble vitamin contents

To optimize the components of *Ganoderma lucidum* (GL) extract, we measured the amount of β-glucan and vitamin B (Supplementary Table 1). β-Glucan content of GL extract was measured using Yeast and Mushroom β-glucan Assay Kit (Megazyme Ltd., Bray, Wicklow County, Ireland) according to the manufacturer's instructions. Briefly, the dried mushroom samples (100 mg) were weighed into culture tubes, and 1.5 ml of concentrated HCl (37%) was added. After heating at 30°C for 45 min, 10 ml of distilled water was added, and the samples were incubated in a boiling water bath for 2 h. After a neutralization step with 2 M KOH, the samples were adjusted to 100 ml with a sodium acetate buffer (pH 5.0). 0.1 ml of aliquots were mixed with exo-1,3-beta-glucanase (20 U/ml) and beta-glucosidase (4 U/ml) and incubated in a water bath at 40°C for 60 min. Then, 3 mL of glucose-oxidase-peroxidase-reagent (GOPOD) was added and again incubated at 40°C for 20 min. β-Glucan content was measured at 510 nm wavelength using spectrophotometer.

Standards for soluble vitamins, thiamine, riboflavin and niacin were purchased from Sigma-Aldrich Co. (St. Louis, MO, USA). Vitamin contents were measured using HPLC and fluorescence and photodiode array (Agilent Co., Santa Clara, CA, USA). HPLC analysis of GL extract was performed as 0.5-1.0 ml/min of flow rate at 35-40°C using Capcell-pak C18 (4.6×150 mm, 5 μm) or capcell-pak C18 MG (4.6×250 mm, 5 μm) columns. 10 mM NaH_2PO_4/0.15 M $NaClO_4$ in H_2O, 10 mM NaH_2PO_4 in H_2O and 5 mM sodium hexanesulfonate/0.1% acetic acid in H_2O were used as the mobile phase buffers for vitamin

B1, B2 and B3, respectively. Wavelengths for detecting vitamin B1, B2 and B3 were 450 nm, 530 nm and 260 nm, respectively.

Table S1. β-Glucan and soluble vitamins contained in *Genoderma Lucidum* extract.

Components	Contents
β-Glucan (g/100 g)	34.15 ± 5.45
Soluble vitamin (mg/100 g)	
Vitamin B_1	0.23 ± 0.01
Vitamin B_2	0.70 ± 0.04
Vitamin B_3	1.78 ± 0.09
Total	2.71 ± 0.11
